# The Influence of Psychological Capital and Social Capital on the Entrepreneurial Performance of the New Generation of Entrepreneurs

**DOI:** 10.3389/fpsyg.2022.832682

**Published:** 2022-05-09

**Authors:** Ruoqi Wang, Haijun Zhou, Lei Wang

**Affiliations:** ^1^School of Information and Intelligent Engineering, Zhejiang Wanli University, Ningbo, China; ^2^Zhejiang Security Vocational and Technical College, Wenzhou, China

**Keywords:** psychological capital, social capital, entrepreneurial performance, new generation of entrepreneurs, deep learning

## Abstract

To enable that the new generation of entrepreneurs can effectively use their own qualities and abilities to improve the level of entrepreneurial performance, and to successfully pass through the entrepreneurial period of the enterprise and achieve longer-term development, the new generation of entrepreneurs is taken as the research object, and firstly, the definition and interaction of psychological capital and entrepreneurial performance are pointed out. Secondly, the questionnaires are designed with reference to the relevant maturity scales, and the reliability, validity analysis, correlation analysis, and multiple linear regression analysis of the collected effective questionnaires are carried out through SPSS and AMOS software. Thirdly, on this basis, it verifies the influence of psychological capital of the new generation entrepreneurs on entrepreneurial performance and the moderating effect of entrepreneurial environment perception. The results show that there is a significant positive correlation between the dimensions of psychological capital and entrepreneurial performance. Gender, age, educational background, marital status, the establishment time of enterprises, and the number of employees all have a significant impact on the psychological capital of the new generation of entrepreneurs. Among them, the psychological capital of the new generation of entrepreneurs aged 31–35 is the best, followed by aged 26–30, 21–25, and the worst is 20 years old and below, which is logical. The correlation coefficient between psychological capital and entrepreneurial performance, social capital and entrepreneurial performance shows a significant positive correlation. The research on the relationship between psychological capital, social capital, and entrepreneurial performance of new generation entrepreneurs systematically explains why some enterprises created by the new generation of entrepreneurs can achieve rapid growth and deepen the research in related fields.

## Introduction

The rapid development of science and technology has promoted the prosperity of the economy and the development of different industries. A large number of entrepreneurs have emerged from all walks of life. Taking “science and technology leading economical development” as a spirit, social entrepreneurs have greatly promoted the further development of the economy of a region and the whole country to create more updated employment opportunities. Nowadays, entrepreneurial economy has become the main engine of global economic growth (Miao et al., 2019; Stefanis, 2020). With the increasing demand for entrepreneurship, entrepreneurial activities are continuously emerging in society. Therefore, research on entrepreneurship research has become a very active research field in universities globally, covering academic fields, such as economics, sociology, business administration, and science (Balboni et al., 2019; Silva et al., 2020). Researchers in every field have their unique research perspectives and have made great achievements in studying entrepreneurship.

Entrepreneurship can promote sustainable economic growth and play a significant important role in economic development. In the current research, the research on entrepreneurship has shifted from the trait theory to the cognitive perspective (Oukes et al., 2019). Scholars have been searching for the factors that affect entrepreneurs’ entrepreneurial ability and success. Different people choose different research directions from which they get their different answers. However, many studies have pointed out that improving a company’s psychological capital is actually very important to the success of entrepreneurship (Lukes et al., 2019). Numerous studies have shown that improving the psychological capital of entrepreneurs is indeed of great significance to successful entrepreneurship (Osman et al., 2019; Salerno, 2019). Psychological capital first appeared in the research in 1997, after that, some scholars analyzed economic capital, human capital, and social capital in entrepreneurship research, emphasized people’s positive psychological strength from the aspects of positive psychological behavior and positive organization, and put forward the concept of “positive psychological capital” (Iazzolino et al., 2019). Experts and scholars are constantly studying and deepening this concept. Psychological theory has gradually become an important research topic in the field of active organizational behavior.

To sum up, although the important influence of psychological capital of entrepreneurs on entrepreneurial performance in the entrepreneurial context is undeniable, the impact mechanism of psychological capital of entrepreneurs on entrepreneurial performance is still unknown. Therefore, the main research purpose is to construct a theoretical model based on the analysis of the psychological capital, social capital, and entrepreneurial performance of the new generation of entrepreneurs, and to effectively measure the psychological capital, social capital, and entrepreneurial performance of the new generation of entrepreneurs. From both direct and indirect perspectives, the influence of psychological capital and social capital on the entrepreneurial performance level of the new generation of entrepreneurs is analyzed, and then, relevant countermeasures and suggestions are proposed.

## Literature Review

Every new technological revolution will bring a new wave of entrepreneurship. Those founders and partners who can seize favorable opportunities and strive to obtain economic value can be regarded as entrepreneurs. Throughout the domestic and foreign literature on the definition of the concept of the new generation of entrepreneurs, there is no authoritative and unified definition. It is well known that the new generation is divided based on sociology and demography. At present, the most common definition of “new generation of employees” is the employees born in the 1980s and 1990s, indicating that age boundaries are the most common basis for dividing this new generation group. Nowadays, foreign scholars Tkacz (2016) defined entrepreneurs aged 35 and below as the new generation of social entrepreneurs, while domestic scholars Zhao and Xu (2015) defined the new generation of entrepreneurs as entrepreneurs born after 1985, indicating that the “post-85 s” may indeed be an age boundary for the new generation of entrepreneurs. Lei (2018) concluded that the psychological capital of entrepreneurs has a greater impact on entrepreneurial performance through the study of the relationship between the university’s psychological capital of entrepreneurs and its entrepreneurial performance. Chen et al. (2019) proposed that psychological capital is an important psychological resource, and using 446 new generations of migrant workers as a research sample, the structural equation model was used to analyze the relationship between psychological capital of new generation of migrant workers and their entrepreneurial performance level had a significant positive effect. By reviewing relevant studies, it showed that psychological capital mainly plays a positive role in entrepreneurial performance. Li (2018) analyzed the entrepreneurial data of migrant workers in 13 cities in eastern China and found that China should build a social capital generation and accumulation mechanism for migrant workers in relevant cities as soon as possible, to promote the improvement of the urban entrepreneurial performance of migrant workers. Yang and Wang (2019) used the survey data related to the entrepreneurship of immigrants in the Three Georges Reservoir and concluded that social capital significantly promoted the improvement of the entrepreneurial performance of immigrants. Ma and Wang (2018) used the dual perspectives of social capital and psychological capital to propose the influence of social capital and psychological capital on the entrepreneurial performance of migrant workers. The results showed that social capital and psychological capital are positively correlated with the entrepreneurial performance level of migrant workers. Barba-Sánchez and Atienza-Sahuquillo (2017) implemented a theoretical model based on expectation theory to explain the motivation and ability of individual entrepreneurs. The results indicated that expectations, tools, and value of expectations could enhance entrepreneurial motivation. From the review of relevant studies, they showed that there were relatively few studies on the influence of psychological capital and social capital on entrepreneurial performance. Social capital is a productive resource that can promote cooperation among the new generation of entrepreneurs. Good psychological capital can help to improve the social skills of entrepreneurs, and it is also of great help for entrepreneurs to improve their entrepreneurial performance. And scholars generally believe that the social capital of entrepreneurs significantly promotes the improvement of their entrepreneurial performance. However, there is no consensus on how the various dimensions of more specific social capital affect the various dimensions of entrepreneurial performance, and scholars need to conduct more in-depth research in the future.

## Relevant Theories and Methods

### The Concept of Psychological Capital

Many studies have proved that psychological factors will affect enterprise performance. Wu and Wu (2019) pointed out in the research that the results showed that the positive emotions of employees play an intermediary role in the positive role of supervisors in expressing the positive emotions of employees’ work engagement; work engagement will promote the positive influence of employees’ positive emotions on their innovative behavior. Employees’ negative emotions will promote the influence of supervisors’ negative emotions on employees’ superficial behavior and innovative behavior. Introduction is made to the concept of psychological capital, and discussion is focused on the influence of psychological capital in the process of starting a business (Albright, 2019; Mas and Paoloni, 2019). According to different forms of development, capital can be divided into three types, namely, economic capital, human capital, and social capital. As the name implies, economic capital refers to tangible assets, such as capital and property, owned by people. Human capital refers to the total value of labor (including physical labor and mental labor) attached to human body’s physical strength and intelligence (Chambers et al., 2019; Aagaard et al., 2020). Social capital refers to the association between individuals or groups-social network, reciprocal norms, and the resulting trust. It is the sum of resources brought by people’s position in the social structure.

**Figure 1:**
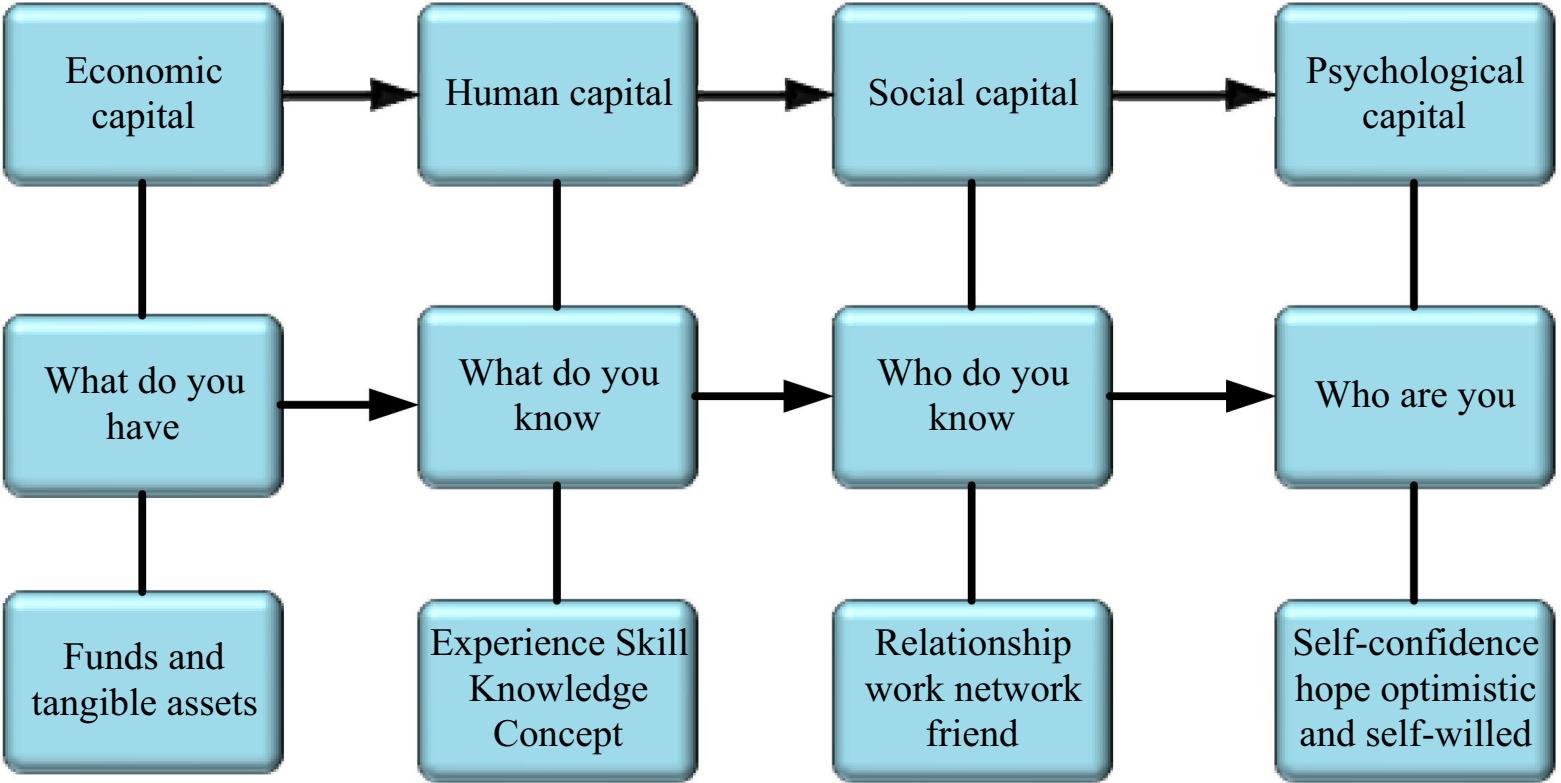
Four major capital forms.

In the existing research, there are many literatures that focus on the influence of economic capital, human capital, and social capital on work and entrepreneurship. However, more and more managers and scholars realize that with the rapid development of global economy, the competition among enterprises is also intensifying. In the past, only through the traditional human capital and social capital for investment and development, it is not enough to maintain the strong competition of enterprises (Alexander and Fry, 2019; Webber et al., 2020). Therefore, there are more and more studies on positive psychological resources, and many empirical research results have been obtained. Figure 1 shows the four capitals recognized by academia to enhance the competitive advantages of individuals and organizations.

The structure and meaning of psychological capital strengthen and supplement each other, namely, they play a complementary role (Badakhshian and Samiee, 2020). On one hand, strengthening the scientific connotation of psychological capital can play an auxiliary role in improving the structure of psychological capital; on the other hand, supplementing and optimizing the structure of psychological capital can play a feedback role in understanding the connotation of psychological capital, thereby developing more tools for measuring psychological capital.

At present, there is no unified conclusion about the composition of psychological capital, but in theory, the definition of psychological capital mainly involves four aspects, namely, self-efficacy, optimism, hope, and resilience (Anglin et al., 2018; Sherman, 2019). The development and definition of psychological capital theory focuses on three main aspects: trait theory, state theory, and integration theory.

### Concepts Related to Entrepreneurial Performance

In many current studies, there is no clear definition of entrepreneurial performance. But through summarizing previous studies, it can be found that entrepreneurial performance is usually evaluated and measured by the impact of new enterprises. Different researchers have given different definitions of entrepreneurial performance (Harms et al., 2018). Some researchers believe that the study of entrepreneurial performance must consider the social and internal environment closely related to entrepreneurial success. Some researchers believe that entrepreneurial achievements are various and are the result of an organization reaching a certain degree. Entrepreneurial performance is systematic. Only by starting from three aspects: entrepreneurship, entrepreneurial team, and entrepreneurial environment can scholars find suitable indicators. When entrepreneurs participate in entrepreneurial activities, they will show a lot of entrepreneurial behavior. These entrepreneurial behaviors should be regularly monitored and evaluated, because only the correct entrepreneurial behavior can produce good results (Kang and Busser, 2018; Obschonka et al., 2020). Generally speaking, entrepreneurial performance is a collection of the total achievements and results achieved by entrepreneurial organizations (Linton and Kask, 2017; Pan et al., 2019). Entrepreneurial performance of a new generation of entrepreneurs is a general term for the achievements and efficiency that new enterprises can achieve in the entrepreneurial process, and is one of the most important indicators of entrepreneurial theory (Miller et al., 2019; Desai and Dearmond, 2021). There are three common indicators for monitoring entrepreneurial performance, which are financial indicators, performance indicators, and objective indicators. Financial indicators are an intuitive way to measure performance, and data are easy to obtain. Sales growth, return on investment, return on assets, price, and other effective information can be obtained through the company’s financial statements. However, when data are applied to specific research, it will be adjusted according to actual needs.

Performance indicators are factors used to determine the performance of entity objects. It is an important part of performance appraisal task, which is reflected in behavior index and result index. Behavioral indicators refer to employees’ behavior in the workplace, while performance indicators refer to specific changes in work and organization due to workers’ work (Qasim and Jader, 2021). Key performance indicators, also known as key performance indicators (KPIs), are an objective quantitative management indicator, which measures process performance by defining, sampling, calculating, and analyzing key parameters of internal process input and output.

### Application of Psychological Capital Theory

As the promoter and main participant of entrepreneurial activities, entrepreneurs have great influence on entrepreneurial performance. The research on entrepreneur personality paradigm is widespread in related fields, but there are few consistent conclusions (Arias et al., 2018; Zhou et al., 2019). It is difficult to describe entrepreneurship in the general sense with stable personal characteristics. Therefore, many researchers transform these personal characteristics. Self-efficacy, the ability to seize the opportunity, cognitive motivation, and other concepts are used to describe the characteristics of a people. Psychological capital, economic capital, human capital, and social capital are the factors that jointly promote the success of enterprises, while psychological capital controls the extended capital model that provides competitive advantage. Someone can achieve greater success as an entrepreneur by better adjusting economic, human, and social capital. Entrepreneurial performance is the general name of entrepreneurial organization performance. Researchers still face many challenges in measuring entrepreneurial performance. It is difficult to obtain objective and reliable entrepreneurial performance data, and it is difficult to compare and analyze several research results in detail. However, the social capital of the new generation of entrepreneurs is generally recognized in significantly improving enterprise performance, so social capital has become an important predictor of enterprise performance. While, there is no consensus on how different aspects of social capital will affect entrepreneurial performance. As the selected intermediate variables or adjustment variables are limited. Therefore, further analysis and research should be carried out on related intermediate variables or regulatory variables (Barba-Sánchez et al., 2022).

### Theoretical Models and Assumptions

**Figure 2:**
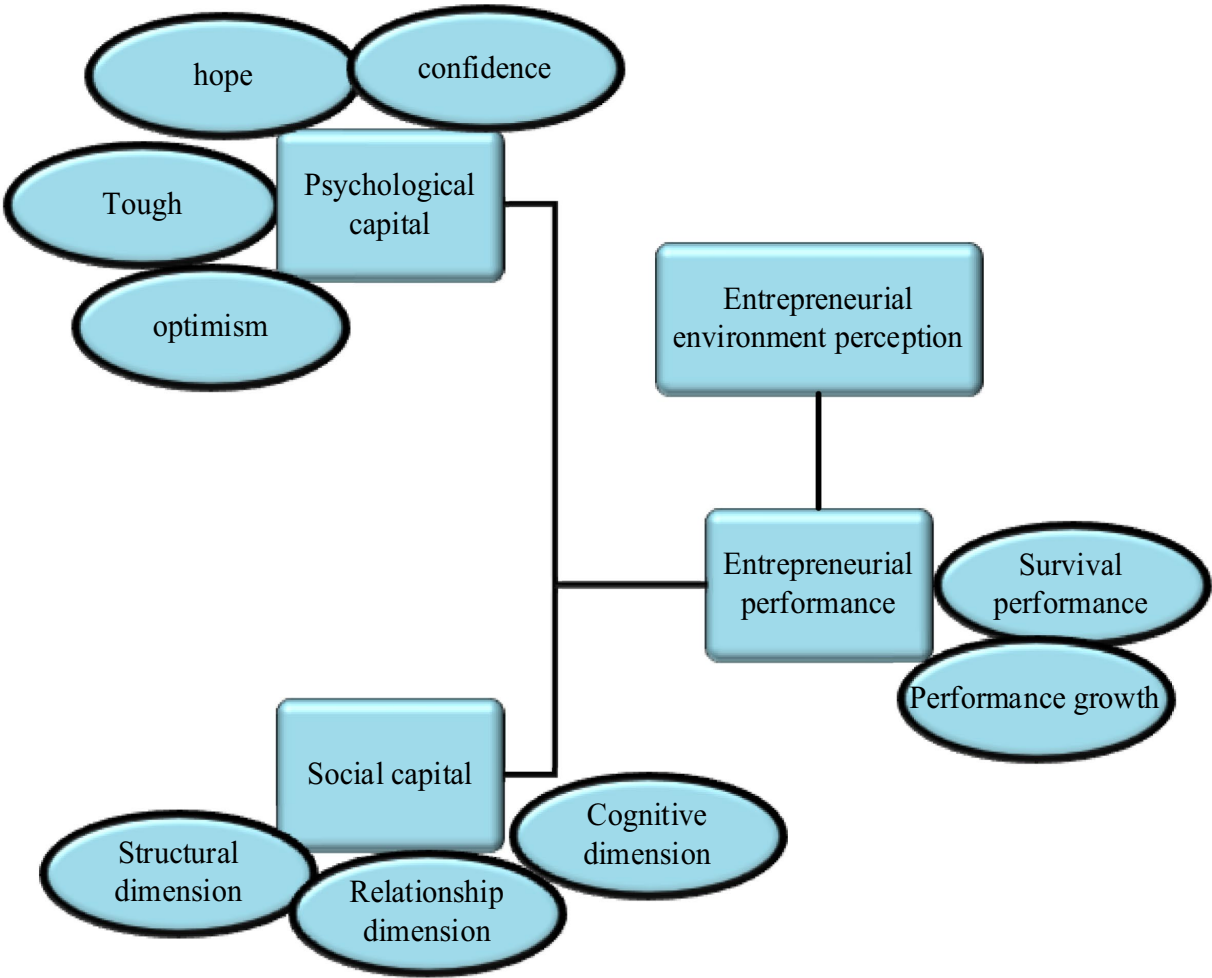
Research model of psychological capital, social capital, and entrepreneurial performance.

Based on the theory of psychological and social capital, a theoretical research model of psychological and social capital mechanism for entrepreneurial performance of new generation entrepreneurs is constructed from three aspects: research level, path, and main factors. The research level has two parts: psychological capital and social capital. The research path is the main influence of psychological and social capital on entrepreneurial performance. The perception of entrepreneurial environment lies in the slow release of psychological and social capital’s influence on corporate performance. The main factors include psychological capital and social capital. Figure 2 displays the theoretical model.

The variables contained in the model are as:

Independent variable: psychological capital: self-confidence, hope, tenacity, and optimism. Social capital: structure, relationship, and perception.

Alternative variables: entrepreneurial awareness, entrepreneurial culture awareness, entrepreneurial incentive awareness, entrepreneurial support awareness, etc.

Knowledge: market network knowledge and non-market network knowledge.

Dependent variables: Entrepreneurial performance has two aspects: survival performance and growth performance.

Control variables: including gender, age, education level, marital status, previous entrepreneurial experience, industry, establishment time, and number of employees.

### Research Hypothesis

The theory of psychological capital emphasizes a positive psychological force of individuals. The concept of psychological capital is derived from positive organizational behavior. Positive organizational behavior is the study and application of human resource advantages and mental abilities that are positively oriented, measurable, developable, and effectively manageable to improve workplace performance. Positive psychological states, such as self-confidence, hope, resilience, and optimism, have become the main research objects of positive organizational behavior because they meet their standards. Therefore, academics generally agree that psychological capital is mainly composed of four dimensions: self-confidence, hope, resilience, and optimism. On the basis of this, studying and applying the psychological capital advantages and abilities of the new generation of entrepreneurs can also improve their entrepreneurial performance. Among them, self-confidence refers to the belief that oneself adjusts cognitive patterns and takes actions to accomplish work goals. Confidence, hope, resilience, and optimism in the psychological capital of entrepreneurs can significantly promote the development of various entrepreneurial activities. Psychological capital helps to improve entrepreneurs’ social ability and communication skills, which is also of great help for entrepreneurs to improve their entrepreneurial performance. Based on the above theoretical analysis, it is inferred that entrepreneurs with positive psychological capital have significant psychological advantages in determining entrepreneurial goals, exploring entrepreneurial opportunities, overcoming various entrepreneurial difficulties, stimulating the entrepreneurial potential of the organization, etc. This psychological advantage will promote entrepreneurs to carry out various entrepreneurial activities, thereby enhancing their entrepreneurial performance.

Therefore, it is hypothesized as:

*H1:* Psychological capital has a significant positive effect on entrepreneurial performance.

*H1a:* Confidence has a significant positive effect on survival performance.

*H1b:* Hope has a significant positive effect on survival performance.

*H1c:* Resilience has a significant positive effect on survival performance.

*H1d:* Optimism has a significant positive effect on survival performance.

*H1e:* Confidence has a significant positive effect on growth performance.

*H1f:* Hope has a significant positive effect on growth performance.

*H1g:* Resilience has a significant positive effect on growth performance.

*H1h:* Optimism has a significant positive effect on growth performance positive influence.

Social capital helps the new generation of entrepreneurs to obtain the resources and information needed for entrepreneurship, helps to promote the success of entrepreneurship, and then improves their entrepreneurial performance.

Therefore, it is hypothesized as:

*H2:* Social capital has a significant positive impact on entrepreneurial performance.

*H2a:* Structural dimension has a significant positive impact on survival performance.

*H2b:* Relationship dimension has a significant positive impact on survival performance.

*H2c:* Cognitive dimension has a significant positive impact on survival performance.

*H2d:* Structural dimension has a significant positive impact on growth performance.

*H2e:* Relationship dimension has a significant positive impact on growth performance.

*H2f:* Cognitive dimension has a significant positive impact on growth performance.

Entrepreneurs perceive different entrepreneurial environments, and psychological capital has different effects on their entrepreneurial performance. In a good entrepreneurial environment, the role of social capital will decrease for entrepreneurs, while the impact of psychological capital on entrepreneurial performance will increase. This is because a good entrepreneurial environment is conducive to the accumulation of psychological capital. On a good material basis, the role of a positive psychological state is particularly obvious. The better the psychological capital, the higher the level of entrepreneurial performance. On the contrary, in a poor entrepreneurial environment, the urgent need for entrepreneurial capital of basic material will greatly enhance the role of social capital. Meanwhile, the entrepreneurial environment is not conducive to the accumulation of psychological capital and may even reduce psychological capital, and the significance of psychological capital is also greatly weakened.

Therefore, it is hypothesized as:

*H3:* Perception of the entrepreneurial environment plays a moderating role between psychological capital and entrepreneurial performance. The better the perception of the entrepreneurial environment, the stronger the relationship between psychological capital and entrepreneurial performance.

*H3a:* Perception of the entrepreneurial environment plays a moderating role between self-confidence and survival performance, and the better the perception of the entrepreneurial environment, the stronger the effect.

*H3b:* Perception of the entrepreneurial environment plays a moderating role between hope and survival performance. The better the perception of the entrepreneurial environment, the stronger the effect.

*H3c:* Perception of the entrepreneurial environment plays a moderating role between resilience and survival performance, and the better the perception of the entrepreneurial environment, the stronger the effect.

*H3d:* Perception of the entrepreneurial environment plays a moderating role between optimism and survival performance. The better the perception of the entrepreneurial environment, the stronger the effect.

*H3e:* Perception of the entrepreneurial environment plays a moderating role between self-confidence and growth performance. The better the perception of the entrepreneurial environment, the stronger the effect.

*H3f:* Perception of the entrepreneurial environment plays a moderating role between hope and growth performance. The better the perception of the entrepreneurial environment, the stronger the effect.

*H3g:* Perception of the entrepreneurial environment plays a moderating role between resilience and growth performance, and the better the perception of the entrepreneurial environment, the stronger the effect.

*H3h:* Perception of the entrepreneurial environment plays a moderating role between optimism and growth performance. The better the perception of the entrepreneurial environment, the stronger the effect.

The entrepreneurial environment perceived by entrepreneurs is different, and the degree of influence of social capital on their entrepreneurial performance will also be different. When perceiving a relatively complete entrepreneurial environment, such as policies, finance, commerce, hardware facilities, services, and culture, entrepreneurs can usually obtain the basic material and capital required for starting a business relatively easily; simultaneously, the dependence on social capital will be less. Conversely, in a poor perception of the entrepreneurial environment, entrepreneurs often have to strengthen social capital to better obtain the basic material and capital required for their own business, and their dependence on social capital will be greater. Therefore, favorable perception of the entrepreneurial environment plays a negative moderating role in the impact of social capital on entrepreneurial performance.

Therefore, it is hypothesized as:

*H4:* Perception of the entrepreneurial environment plays a moderating role between social capital and entrepreneurial performance. The better the perception of the entrepreneurial environment, the weaker the relationship between social capital and entrepreneurial performance.

*H4a:* Perception of the entrepreneurial environment plays a moderating role between the structural dimension and survival performance. The better the perception of the entrepreneurial environment, the weaker the effect.

*H4b:* Perception of the entrepreneurial environment plays a moderating role between relational dimension and survival performance, the better the perception of the entrepreneurial environment, the weaker the effect.

*H4c:* Perception of the entrepreneurial environment plays a moderating role between cognitive dimension and survival performance. The better the perception of the entrepreneurial environment, the weaker the effect.

*H4d:* Perception of the entrepreneurial environment plays a moderating role between the structural dimension and growth performance. The better the perception of the entrepreneurial environment, the weaker the effect.

*H4e:* Perception of the entrepreneurial environment plays a moderating role between relationship dimension and growth performance, the better the perception of the entrepreneurial environment, the weaker the effect.

*H4f:* Perception of the entrepreneurial environment plays a moderating role between cognitive dimension and growth performance. The better the perception of the entrepreneurial environment, the weaker the effect.

Through comparison, it shows that in different entrepreneurial environments, psychological capital and social capital have different degrees of vital influence on entrepreneurial performance, that is, the entrepreneurial environment has played a moderating role in this process.

### Questionnaire Design

1. Psychological capital questionnaire design
  The research divides psychological capital into four dimensions: confidence, hope, resilience, and optimism. Self-confidence refers to the positive belief in the cognitive model being adjusted and taking actions to achieve the task objectives. It is a positive state based on personal experience and formed by the intersection of success factors and realization paths. Resilience refers to an individual’s ability to recover quickly from adversity, conflict, and frustration. Optimism is a tendency of positive attribution. Some positive situations are attributed to permanence and universality, while some negative events are attributed to temporality.
2. Social capital questionnaire design
  It is believed that social capital has three dimensions: structure, relationship, and cognition. Dimension refers to various types of connections among participants in social activities. Relationship refers to the relationship model produced by people in their interactions. Cognition represents the common goal among different members and cultures. Likert’s 5-point scoring system is adopted to calculate scores from “very different” to “very agree.”
3. Entrepreneurial performance questionnaire
  Entrepreneurial performance questionnaire has two dimensions to measure entrepreneurial performance, namely, survival performance and growth performance, because the characteristic of entrepreneurial performance is that new activities of enterprises must survive before they can develop. Survival items range from “very different” to “very agreed,” with scores ranging from 1 to 5. Growth performance ranges from “far below the industry average” to “far above the industry average,” with scores ranging from 1 to 5.
4. Questionnaires of entrepreneurial environment awareness
  Entrepreneurial environment can be divided into five aspects: enterprise culture, enterprise incentive, enterprise support, market network, and non-market network. Based on the above five aspects, a design is made on the perceptual measure of entrepreneurial environment. Likert’s five-point scoring method is used for the cultural cognition of entrepreneurship, ranging from “very inconsistent” to “very consistent,” with a score ranging from 1 to 5 points.

### Data Sources and Hypothesis Testing

The methods of questionnaire and empirical analysis are used, among which the software of SPSS 23.0 and AMOS 23.0 is used for data analysis. Based on descriptive statistical analysis, Cronbach’s alpha coefficient and validation factor analysis are used to test the reliability and validity of each variable. Then, to understand the relationship between the entrepreneurial performance of enterprises in detail, correlation analysis on psychological capital, social capital, and entrepreneurial performance is carried out.

The questionnaire is distributed in two forms: paper questionnaire and online questionnaire. The new generation of entrepreneurs from multiple entrepreneurial bases fills in the questionnaire (the new generation of entrepreneurs mainly come from Chuangkebang Incubation Base, Jiangning District, Nanjing City, Jiangsu Province, Pioneer Building, High-tech Zone, Jiangning City, Life Science and Technology Town, Nanjing City, E-commerce Industrial Park, Jiangning City, and other entrepreneurial bases). It involves all walks of life, including the Internet, finance, manufacturing, and business services and limits the age of population variables, and the age of the target must be under 35. A total of 500 questionnaires are distributed in this survey, and 472 questionnaires are recovered, with a recovery rate of 94.4%. Among them, 438 valid questionnaires can be used as sample analysis, and the final valid questionnaire rate is 87.6%. The basic information part of the respondents in the questionnaire, including gender, age, education, and marital status, as well as related entrepreneurial information, including the industry to which the enterprise belongs, the time of the establishment of the enterprise, the number of employees of the enterprise, the number of previous entrepreneurial experiences, the main motivation of entrepreneurship, and the factors that are considered to have a greater impact on entrepreneurship. Table 1 shows the descriptive statistical analysis results of the effective recovery of samples.

**Table 1:** Descriptive statistical analysis of samples.

Item	Category	Number of People	Percentage (%)
Gender	Male	225	51.4
	Female	213	48.6
Age	20 years old and below	57	13.0
	21–25 years old	80	18.2
	26–30 years old	71	16.3
	31–35 years old	230	52.5
Education	High school and below	14	3.3
	college	55	12.5
	Undergraduate	221	50.5
	Graduate and above	148	33.7
Marital status	Married	247	56.5
	Unmarried	191	43.5
Industry the enterprise belongs to	Agriculture, Forestry, Animal Husbandry, Fishing	4	0.8
	Mining industry	4	0.8
	Manufacturing	40	9.2
	Electricity, heat, etc.	8	1.9
	Construction industry	15	3.5
	Wholesale and retail trade	18	4.1
	Transportation	21	4.9
	Accommodation and catering industry	11	2.4
	Computer	78	17.7
	Finance	42	9.5
	Scientific research	14	3.3
	Public Utilities	2	0.5
	Service	11	2.4
	Real estate	13	3.0
	lease	10	2.2
	Health care	12	2.7
	Education	53	12.0
	Culture and Sports	44	10.1
	other	38	9.0
The time of the establishment of the enterprise	Within 1 year	69	15.7
	1–3 years	111	25.3
	3–5 years	57	13.0
	5–10 years	53	12.0
	10+ years	148	34.0
The number of employees of the enterprise	10 people or less	136	31.0
	11–50 people	83	19.0
	51–100 people	48	10.9
	101–200 people	30	6.8
	More than 200 people	141	32.3
The number of entrepreneurial experiences	0	274	62.5
	1 time	94	21.5
	More than 2 times	70	16.0
The motivation of entrepreneurship	Improve living quality	113	25.8
	Improve quality of life	7	1.6
	Realize personal ideals	263	60.1
	Conform to the trend of social development	20	4.6
	Other	35	7.9
Influencing factors of entrepreneurship	Home and school	56	12.8
	Optimism	42	9.5
	Social support	56	12.8
	Self-confidence	45	10.3
	Entrepreneurial team	173	39.4
	Tough character	46	10.6
	Other	20	4.6

## Questionnaire Survey and Analysis Results

### Pre-survey Results of Questionnaire

**Figure 3:**
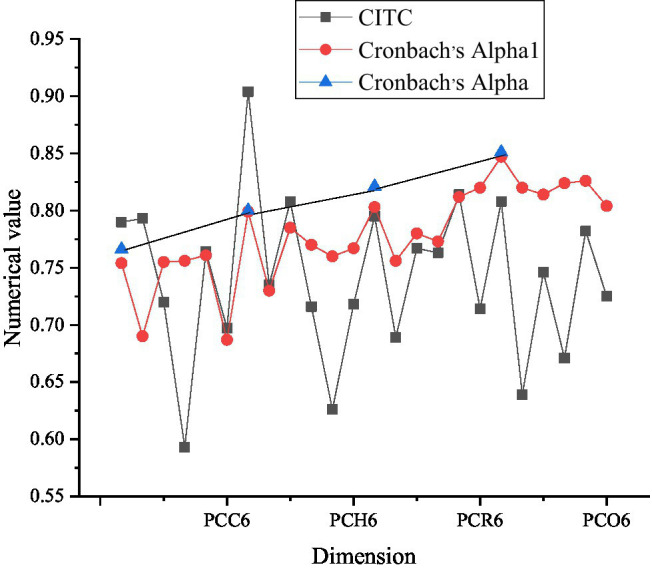
Reliability analysis of psychological capital (PCC6 represents six evaluation factors of psychological capital confidence dimension, PCH6 represents six evaluation factors of hope dimension, PCR6 represents six evaluation factors of tenacity dimension, and PCO6 represents six evaluation factors of optimism dimension).

Questionnaires are collected and selected before the survey. They are analyzed to confirm the reliability. Cronbach’s alpha factor is selected for testing. Figure 3 shows the results of psychological capital reliability analysis.

As Figure 3 signifies, according to the corrected item-total correlation (CITC) value, the values of confidence, hope, resilience, and optimism in the psychological capital scale are all greater than 0.5. Deleting any one of them will lower the Cronbach’s alpha coefficient of the dimension, which indicates that the correlation between these dimensions is relatively high. Cronbach’s alpha values of the four dimensions are 0.766, 0.800, 0.821, and 0.851, respectively. Generally speaking, the higher the coefficient, the higher the reliability of the tool. In basic research, the reliability should be at least 0.80, while in exploratory research, it can be accepted as long as it reaches 0.70. The reliability between 0.70 and 0.98 is high, while the reliability below 0.35 is at a low level. Therefore, the reliability of the four dimensions is very high.

**Figure 4:**
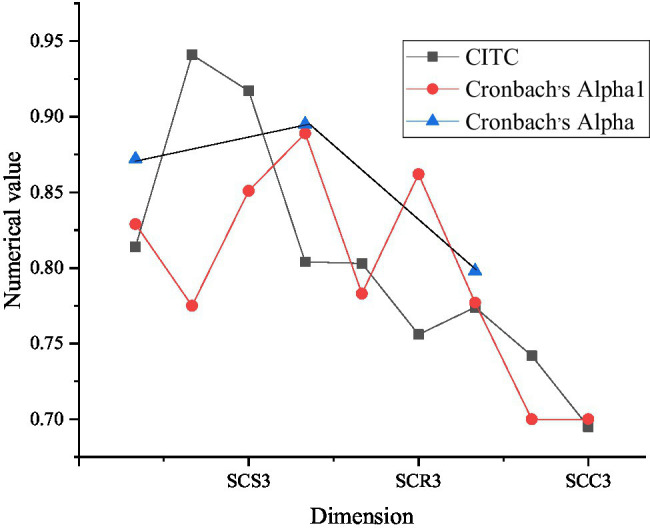
Reliability analysis of social capital (SCS3 represents three evaluation factors of social capital structure dimension, SCR3 represents three evaluation factors of relationship dimension, and SCC3 represents three evaluation factors of cognitive dimension).

Figure 4 denotes the results of social capital reliability analysis.

As Figure 4 illustrates, the social capital scale includes three dimensions: structure, relationship, and cognition, and the CITC values of the items corresponding to these dimensions are all above 0.5, which indicates that the correlation between these dimensions is relatively high. From the overall Cronbach’s alphas coefficient, the total Cronbach’s alphas coefficients of the three dimensions are 0.872, 0.895, and 0.798, respectively, which indicates that the reliability of these three dimensions has reached a relatively high level.

**Figure 5:**
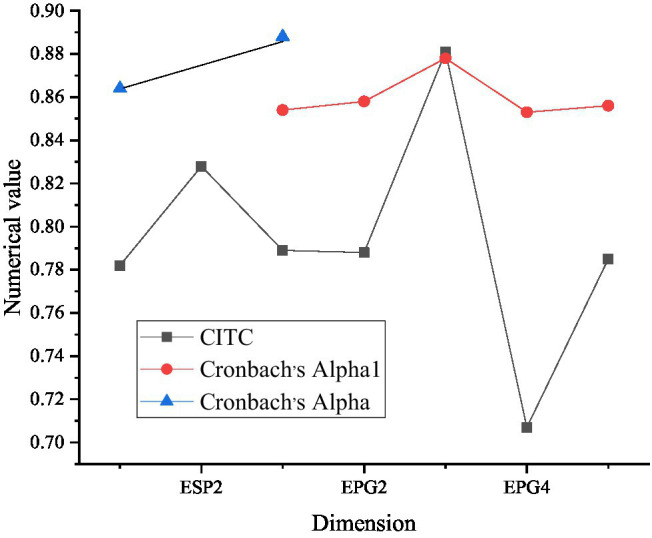
Reliability analysis results of entrepreneurial performance scale (ESP2 represents two evaluation factors of survival performance in entrepreneurial performance, EPG2 represents the first two evaluation factors of growth performance dimension, and EPG4 represents four evaluation factors of growth performance dimension).

Figure 5 implies the reliability analysis results of entrepreneurship performance scale.

As Figure 5 shows, the Entrepreneurship Performance Scale contains only two dimensions, namely, survival performance and growth performance. The CITC values of all factors in the two dimensions are greater than 0.5, and removing any one of them will reduce the Cronbach’s alpha coefficient of the dimension, which means that the correlation between these dimensions is relatively high. Among them, the overall Cronbach’s alpha of survival and growth dimensions are 0.864 and 0.888, respectively. It shows that the two-dimensional scale of entrepreneurial performance has good reliability.

**Figure 6:**
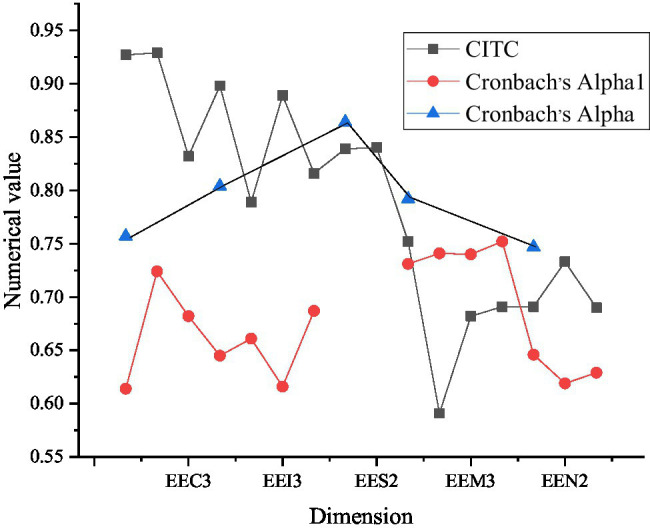
Reliability analysis results of entrepreneurial environment perception scale (EEC3 indicates three evaluation factors of entrepreneurial culture in entrepreneurial environment, EEI3 indicates three evaluation factors of entrepreneurial incentive, EES2 indicates two evaluation factors of entrepreneurial support, EEM3 indicates three evaluation factors of market network, and EEN2 indicates two evaluation factors of non-market network).

Figure 6 reveals the reliability analysis results of entrepreneurship perception scale.

On the scale of entrepreneurial environment awareness, evaluation is made from five aspects: entrepreneurial culture awareness, entrepreneurial incentive awareness, entrepreneurial support awareness, market network awareness, and non-market network entrepreneurial awareness. From the CITC values of these dimensions, all dimensions have reached more than 0.5. When one of the dimension factors is eliminated, the Cronbach’s alpha factor of the dimension will decrease, which shows that the correlation between these factors is relatively high. Overall, Cronbach’s alpha coefficients of the five dimensions are 0.757, 0.804, 0.864, 0.792, and 0.747, respectively. Therefore, the reliability of results is relatively good.

### Analysis Results of Questionnaire Survey Data

**Figure 7:**
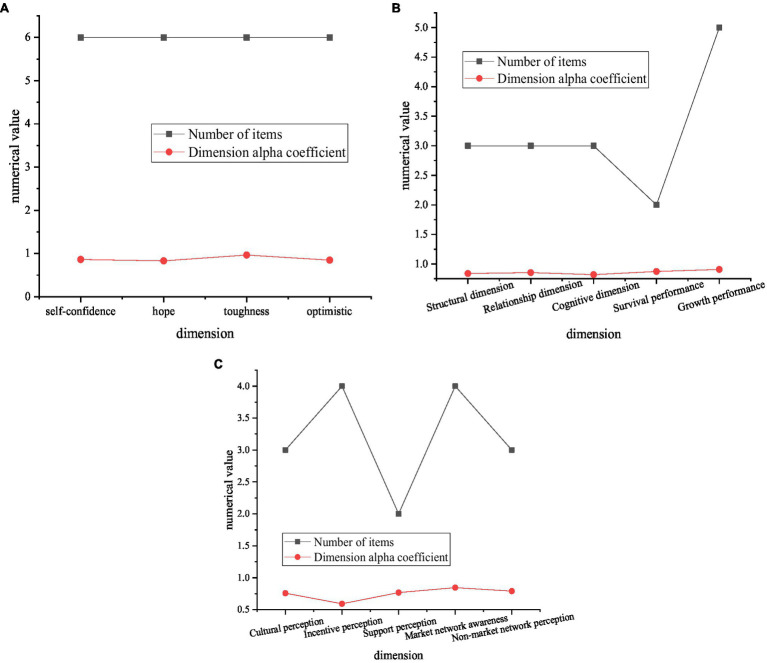
Reliability analysis results of the scale **(A)** shows the reliability analysis results of psychological capital scale, **(B)** shows the reliability analysis results of social capital and entrepreneurial performance scale, and **(C)** shows the reliability analysis results of entrepreneurial environment perception scale.

Figure 7 signifies reliability analysis results of the scale.

According to the reliability analysis results shown in Figure 7, Cronbach’s Alpha coefficients of the four scales are all above 0.8, and the reliability is acceptable as long as it reaches 0.70, and the reliability between 0.70 and 0.98 is high, so the reliability is relatively good and the internal consistency is relatively high. The four-part table can be used herein.

**Figure 8:**
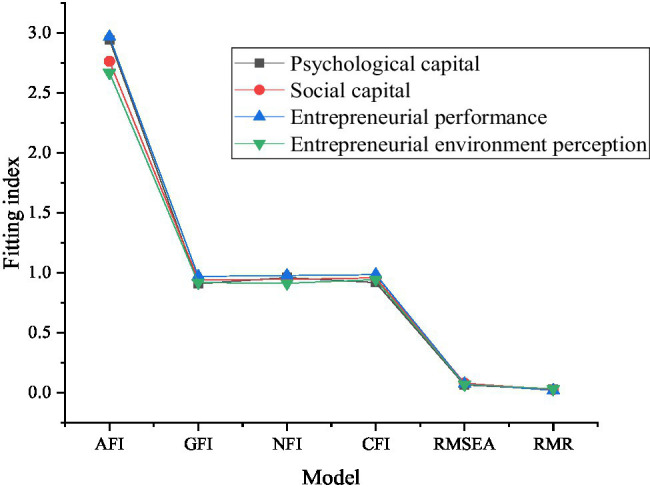
Validity analysis results of the scale (AFI is the absolute fitting index; GFI, NFI, CFI, RMSEA, and RMR are the goodness-of-fit evaluation indexes of AMOS software).

Figure 8 presents the validity analysis results of the four scales.

As Figure 8 presents, the absolute fitting index (^2^/df) of the four groups of scales is less than 3. The fitness of GFI is greater than 0.9, the approximate errors of RMR and RMSEA of RMS residuals are both less than 0.08, and the NFI and CFI after mass relative adjustment are both greater than 0.90, which meet the standards of various indexes. Based on the above indexes, the fitting degree of the model is improved, which proves that the measurement is more effective, and these values are in line with each index standard. Based on the above indexes, it can be proved that the model has good fitting effect and high measurement validity.

**Figure 9:**
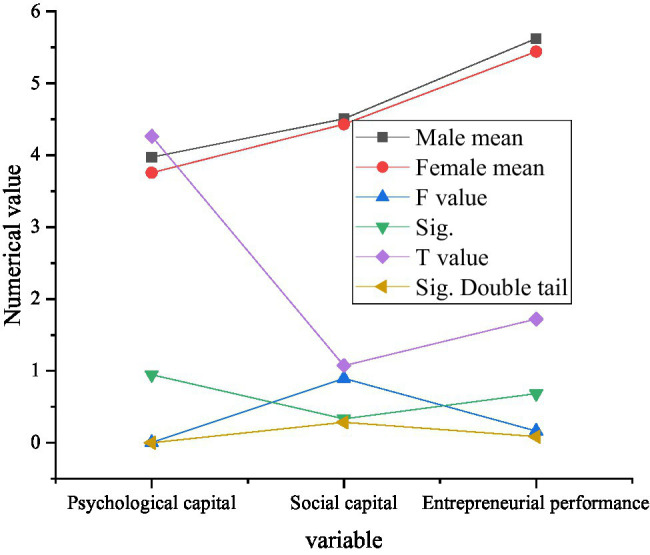
Independent sample *T*-test results.

In order to verify the differences in psychological capital, social capital, and entrepreneurial performance of factors, such as gender, age, educational background, marital status, enterprise establishment time, and number of employees, a design is made on independent sample *T*-test to conduct multiple variable analysis, respectively. Figure 9 displays the results of independent sample *T*-test.

In the independent sample *T*-test results shown in Figure 9, the significant levels of psychological capital of the three variables are all less than 0.05, indicating that there are significant differences in psychological capital between male and female entrepreneurs in the new generation. Relatively speaking, the level of entrepreneurial psychological capital of men is higher than that of women, and the average value of 3.9724 is higher than that of 3.7561 female entrepreneurs. The significant level of social capital and entrepreneurial performance is above 0.05, which indicates that there is no significant difference between social capital and entrepreneurial performance of new generation male and female entrepreneurs.

**Figure 10:**
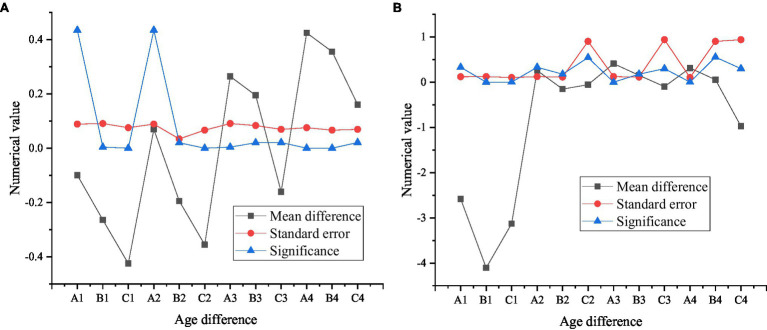
Multiple comparison results of psychological capital and entrepreneurial performance under different ages **(A)** shows the comparison results of psychological capital under different ages; **(B)** shows the comparison results of entrepreneurial performance under different ages, in which A1, B1, and C1 represent 20 years old and below, A2, B2, and C2 represent 21–25 years old, A3, B3, and C3 represent 26–30 years old, and A4, B4, and C4 represent 31–35 years old.

The multiple comparison results of psychological capital and entrepreneurial performance under age differences are shown in Figure 10.

As shown in Figure 10, young entrepreneurs of a certain age group show significant differences in psychological capital and entrepreneurial performance, and the concrete embodiment of the differences can be observed when comparing the average differences. The order of psychological capital of new generation entrepreneurs from high to low is 31–35 years old, 26–30 years old, and 21–25 years old, and entrepreneurs under 20 years old have the lowest psychological capital. But from the perspective of entrepreneurial performance, the new generation of entrepreneurs aged 26–30 perform best as entrepreneurs, followed by entrepreneurs aged 31–35, 21–25, and under 20.

**Figure 11:**
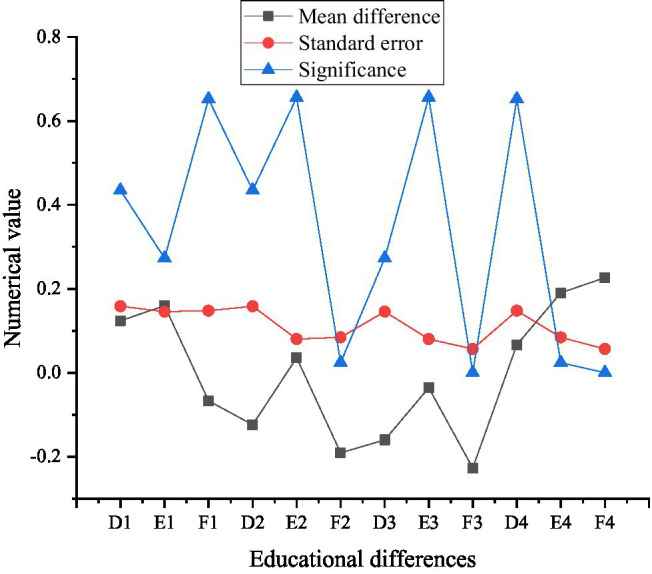
Multiple comparison results of psychological capital under educational differences (D1, E1, and F1 are high school and below, D2, E2, and F2 are college degrees, D3, E3, and F3 are undergraduate degrees, and D4, E4, and F4 are master’s and above).

Figure 11 shows the multiple comparison results of psychological capital under educational background differences.

The results in Figure 11 show whether there are significant differences in psychological capital among specific entrepreneurs from different backgrounds, and the specific differences can be obtained by comparing the average values. From the comparison results in Figure 11, the new generation entrepreneurs with master’s degree or above rank first in psychological capital, second in psychological capital of entrepreneurs with high school education or below, and third in college education. Undergraduate entrepreneurs have the lowest psychological capital.

**Figure 12:**
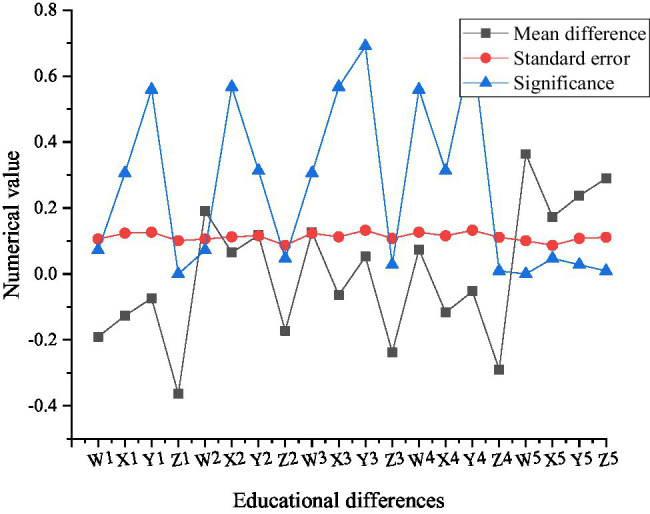
Multiple comparison results of psychological capital of new generation entrepreneurs with different enterprise establishment times (In Figure, W1, X1, Y1, and Z1 are within 1 year of enterprise establishment, W2, X2, Y2, and Z2 are 1–3 years of enterprise establishment, W3, X3, Y3, and Z3 are 3–5 years of enterprise establishment, and W4, X4, Y4, and Z4 are 5–10 years of enterprise establishment, W5).

Figure 12 indicates multiple comparisons of psychological capital of new generation entrepreneurs with different establishment times.

As shown in Figure 12, when starting a business, the new generation entrepreneurs set up companies with significant differences in entrepreneurial performance according to different time groups and find out the specific differences by comparing the average differences. From the comparison results, it can be seen that the new generation of entrepreneurs who have been in business for more than 10 years have the best entrepreneurial performance, and those who have been in business for 1–3 years rank second. Enterprises with a start-up time of 3–5 years rank third, those with a start-up time of 5–10 years rank fourth, and those with a start-up time of 1 year or less rank last.

**Figure 13:**
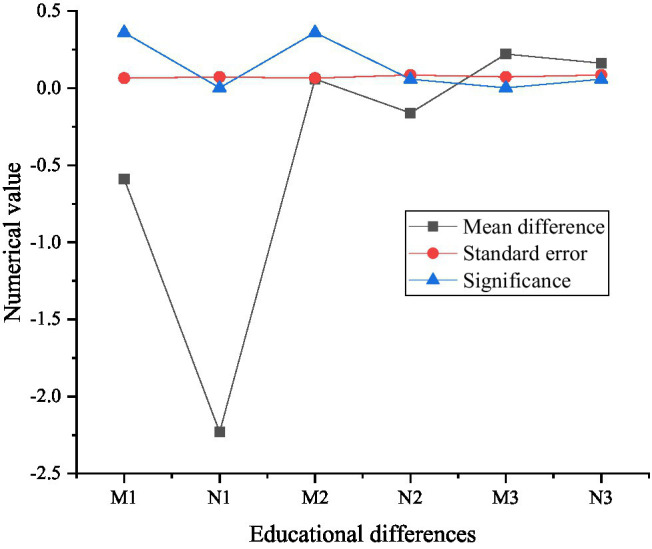
Multiple comparison of entrepreneurs’ entrepreneurial times (in the figure, M1 and N1 represent zero entrepreneurial times, M2 and N2 represent 1–2 entrepreneurial times, and M3 and N3 represent 2 or more entrepreneurial times).

Figure 13 reveals a multiple comparison of the number of entrepreneurs.

Figure 13 signifies the specific differences of psychological capital of new generation entrepreneurs among groups with different entrepreneurial experiences in the past, and the specific differences can be observed by comparing the average differences. Among them, the new generation entrepreneurs with two or more entrepreneurial experiences have the highest psychological capital, followed by entrepreneurs with one entrepreneurial experience, and the worst is 0. Clearly, the experiences of more entrepreneurs in the new generation of entrepreneurs enrich their experiences. Make the psychological capital reach a higher level.

After verifying the reliability and validity of the scale, this paper adopts Pearson correlation analysis, using four dimensions of psychological capital, three dimensions of social capital, and two dimensions of entrepreneurial performance. Firstly, the relationship between two dimensions is investigated, and the correlation between variables is analyzed. Table 2 lists the analysis results by using SPSS 23.0 statistical software.

**Table 2:** Results of Pearson analysis of relations.

Variable	PC	Confidence	Hope	Toughness	Optimism	SC	S1	R	C1	E	S	G
PC	1											
confidence	0.795^**^	1										
hope	0.892^**^	0.630^**^	1									
toughness	0.898^**^	0.594^**^	0.746^**^	1								
optimism	0.86^**^	0.523^**^	0.689^**^	0.748^**^	1							
SC	0.591^**^	0.471^**^	0.534^**^	0.470^**^	0.566^**^	1						
S1	0.442^**^	0.344^**^	0.417^**^	0.363^**^	0.399^**^	0.810^**^	1					
R	0.565^**^	0.469^**^	0.488^**^	0.444^**^	0.550^**^	0.847^**^	0.506^**^	1				
C1	0.468^**^	0.362^**^	0.426^**^	0.365^**^	0.462^**^	0.839^**^	0.509^**^	0.601^**^	1			
E	0.338^**^	0.301^**^	0.309^**^	0.307^**^	0.343^**^	0.407^**^	0.365^**^	0.309^**^	0.342^**^	1		
S	0.388^**^	0.308^**^	0.330^**^	0.324^**^	0.379^**^	0.429^**^	0.330^**^	0.364^**^	0.377^**^	0.844^**^	1	
G	0.376^**^	0.382^**^	0.386^**^	0.327^**^	0.394^**^	0.352^**^	0.382^**^	0.352^**^	0.395^**^	0.834^**^	0.408^**^	1

*** *p < 0.001;*

** *p < 0.01; and*

* *p < 0.5*.

*In the table, PC represents Psychological capital, SC represents Social capital, S1 represents Structural dimension, and R represents Relationship dimension. C1 stands for cognitive dimension, E for entrepreneurial performance, S for Survival performance, and G for Growth performance*.

Pearson coefficient ranges from −1 to 1. The sign of numerical value reflects the direction of correlation between variables, and the absolute value of numerical value reflects the degree of correlation between variables. The closer the absolute value is to 1, the higher the degree of correlation between variables. As Table 1, whether as a whole or subdivided into dimensions, psychological capital and entrepreneurial performance, social capital, and entrepreneurial performance are all significantly positively correlated, and the correlation coefficients are all between 0.3 and 0.9, which are highly positively correlated.

The above research shows that gender differences have a significant impact on the psychological capital of the new generation of entrepreneurs. In contemporary society, although both males and females play a very significant role, actually, entrepreneurs of different genders have different psychological states in the process of starting a business and the psychological capital they have to promote their own growth and performance improvement. Male Entrepreneurs have higher psychological capital, because compared with males, females are more psychologically sensitive, more flustered when encountering problems, and have poorer psychological quality. The age difference has a significant impact on the psychological capital and entrepreneurial performance of the new generation of entrepreneurs.

Among them, the new generation of entrepreneurs between the ages of 31 and 35 have the best psychological capital, followed by 26–30 years old, and then 21–25 years old, the worst is 20 years old and below. This distribution is logical. Obviously, the older the new generation of entrepreneurs, the more mature their psychological capital is compared with the younger generation. The new generation of entrepreneurs between the ages of 26 and 30 have the best entrepreneurial performance, followed by 31–35 years old, 21–25 years old again, and the worst 20 year old and below. Compared with the 31–35-year-old group, they are more motivated and energetic, and they are more stable than the younger age group, and their entrepreneurial performance is naturally higher. Educational differences also have a significant impact on the psychological capital of the new generation of entrepreneurs. The correlation of variables between pairs indicates that the proposed theoretical hypothesis H1 is established, that is, the psychological capital of employees has a significant positive impact on their entrepreneurial performance. The four dimensions of psychological capital, self-confidence, hope, resilience, and optimism have certain influences on the two dimensions of entrepreneurial performance, survival, and growth performance, respectively. The results show that the four dimensions have a significant positive impact on the two dimensions of entrepreneurial performance, that is, the improvement of any psychological capital dimension will lead to a significant increase in entrepreneurial performance of the new generation of entrepreneurs. Hypotheses H1a, H1b, H1c, H1d, H1e, H1f, H1g, and H1h are all established. The test results show that the proposed theoretical hypothesis H2 holds, that is, employees’ social capital has a significant positive impact on entrepreneurial performance. It examines the influence of the three dimensions of social capital: the structural dimension, the cognitive dimension, and the relational dimension on the two dimensions of entrepreneurial performance, survival, and growth performance, respectively. The results show that all three dimensions have a significant positive impact on the two dimensions of entrepreneurial performance, that is, the improvement of any social capital dimension will lead to a significant increase in the entrepreneurial performance of the new generation of entrepreneurs. Hypotheses H2a, H2b, H2c, H2d, H2e, and H2f are established. Meanwhile, the influence of various dimensions of social capital on entrepreneurial performance is greater than that of psychological capital. The theoretical hypothesis H3 is partially established, that is, perception of the entrepreneurial environment plays a partial moderating role in the relationship between psychological capital and entrepreneurial performance. Among them, the perception of the entrepreneurial environment has no significant moderating effect on the relationship between psychological capital confidence dimension and survival performance, the relationship between hope dimension and survival performance, resilience dimension and survival performance, optimism dimension, and survival performance. Hypotheses H3a, H3b, H3c, and H3d are not established. The perception of the entrepreneurial environment plays a positive and significant moderating effect on the relationship between the psychological capital self-confidence dimension and the growth performance, the hope dimension and the growth performance, the resilience dimension and the growth performance, and the optimism dimension and the growth performance. The better the perception of the entrepreneurial environment, the stronger the effect. Hypotheses H3e, H3f, H3g, and H3h are established. The theoretical hypothesis H4 is partially established, that is, the perception of the entrepreneurial environment plays a partial moderating role in the relationship between social capital and entrepreneurial performance. Among them, the perception of the entrepreneurial environment plays a significant negative moderating role between the social capital structure dimension and survival performance, and the relationship between the relationship dimension and survival performance. The better the perception of the entrepreneurial environment, the weaker the effect. Hypotheses H4a and H4b are established. However, the perception of the entrepreneurial environment does not have a significant moderating effect on the relationship between the social capital cognitive dimension and survival performance, and hypothesis H4c is established. The perception of the entrepreneurial environment does not have a significant moderating effect on the relationship between social capital structure dimension and growth performance, relationship dimension and growth performance, and hypotheses H4d and H4e are not established. While the perception of the entrepreneurial environment has a significant negative moderating effect on the relationship between social capital cognition and growth performance, and the better the entrepreneurial environment perception, the weaker the effect, hypothesis H4f is established.

## Conclusion

The psychological and social capital of the new generation entrepreneurs are analyzed primarily, entrepreneurial performance is selected as the evaluation result, and entrepreneurial environment is regarded as external adjustment variable. Attention is focused on the demographic data of entrepreneurial variables and factors that can affect the psychological, social, and entrepreneurial performance of the new generation entrepreneurs, which are control variables. Through qualitative and quantitative analysis, study is conducted on the influence of psychological capital and social capital of new generation entrepreneurs on entrepreneurial performance. The results show that factors, such as gender, age, educational background, marital status, and the time of establishment of enterprises, have a significant impact on the psychological capital of the new generation of entrepreneurs. Among them, the new generation entrepreneurs aged 31–35 have the best psychological capital, followed by 26–30, 21–25, and the worst is 20 and below. The correlation coefficient between psychology and entrepreneurial performance, social capital, and entrepreneurial performance is between 0.3 and 0.9, showing a significant positive correlation. Hypothetical research shows that the psychological capital of employees has a significant positive impact on entrepreneurial performance. The social capital of employees has a significant positive impact on entrepreneurial performance. The perception of the entrepreneurial environment plays a partial moderating role between psychological capital and entrepreneurial performance, and it also plays a partial moderating role between social capital and entrepreneurial performance.

Although certain results have been achieved, there are still many shortcomings in the research process, which are summarized as follows. Firstly, in the aspect of variable measurement, all the data of the survey come from the same theme, and all the elements of the survey are conducted in the form of entrepreneurs’ relatively subjective self-assessment, which has certain limitations. Secondly, in the aspect of sample collection, by combining online and offline forms, good results have been achieved. Quantitative support has collected a sufficient number of surveys, but the sample distribution range is too concentrated, and there is room for further optimization. Thirdly, entrepreneurship is a dynamic process. Investigation is only concentrated on the influence of psychological and social capital on entrepreneurial performance in the same period. Unable to consider the relationship between entrepreneurship and time, so the results are not accurate enough. In the future research, the above points will be given more attention to make the research results more valuable and convincing.

## Data Availability Statement

The original contributions presented in the study are included in the article/supplementary material, further inquiries can be directed to the corresponding author.

## Author Contributions

All authors listed have made a substantial, direct, and intellectual contribution to the work and approved it for publication.

## Funding

Education Programme foundation, Education bureau of Ningbo city 202021YGH039. Education Programme Foundation. Project on the Integration of Industry cooperation HX2019129. Project on the Integration of Industry cooperation HX2020162.

## Conflict of Interest

The authors declare that the research was conducted in the absence of any commercial or financial relationships that could be construed as a potential conflict of interest.

## Publisher’s Note

All claims expressed in this article are solely those of the authors and do not necessarily represent those of their affiliated organizations, or those of the publisher, the editors and the reviewers. Any product that may be evaluated in this article, or claim that may be made by its manufacturer, is not guaranteed or endorsed by the publisher.
